# Role of enteral nutrition in the clinical care, management, and quality of life of patients with inflammatory bowel disease

**DOI:** 10.3389/fnut.2026.1759869

**Published:** 2026-04-29

**Authors:** Qian Chen, Qianlan Zhu, Ruidong Chen, Shuang Zhou, Wen Tang

**Affiliations:** Department of Gastroenterology, The Second Affiliated Hospital of Soochow University, Suzhou, China

**Keywords:** Crohn’s disease, enteral nutrition, inflammatory bowel disease, nutrition, ulcerative colitis

## Abstract

**Background:**

Enteral nutrition (EN) is essential for Inflammatory Bowel Disease treatment. This applies mainly to Crohn’s disease (CD) and ulcerative colitis (UC) patients. The study evaluated the impact of EN treatment on clinical, nutritional, inflammatory, and quality of life outcomes in IBD patients. This study aimed to examine the association between EN therapy’s clinical, nutritional, inflammatory, and Quality of Life (QoL) effects on Inflammatory Bowel Disease patients.

**Methods:**

A retrospective study investigated 750 Chinese IBD patients. These patients had 400 CD and 350 UC diagnoses. Medical records provided data. EN formula, administration, calorie density, and duration were studied along with disease activity, nutritional markers including hemoglobin, albumin, and body mass index, and inflammatory indices like erythrocyte sedimentation rate. Validated IBDQ measures quality of life. Clinical remission was defined as a Crohn’s Disease Activity Index (CDAI) score < 150 for CD patients and a Mayo score ≤ 2 (with no individual subscore > 1) for UC patients, assessed after completion of EN therapy. Chi-square and *t*-tests were used for statistical analysis, with a significance level of *p* < 0.05.

**Results:**

Baseline characteristics of 750 patients are comparable between groups. Elemental and semi-elemental formulas were more frequently prescribed in CD patients and were associated with higher caloric and protein intake (*p* < 0.05). Approximately 60% of patients achieved clinical remission after 6 ± 2 weeks of EN therapy (62.5% CD vs. 57.1% UC). Improvements in BMI, albumin, hemoglobin, and CRP were observed in patients receiving EN, along with increased IBDQ scores. Adverse effects were minor (8%). Full adherence (OR = 2.15, *p* < 0.001) and longer EN duration (>6 weeks) were independently associated with higher odds of remission and QoL improvement.

**Conclusion:**

EN therapy corresponded with clinical remission, improved nutritional markers, and reduced inflammation in IBD patients, particularly those with CD. These findings support the potential role of EN as a complementary strategy in IBD management; however, prospective randomized trials are further confirming these associations and standardize therapeutic protocols.

## 1 Introduction

Inflammatory Bowel Disease (IBD), encompassing Crohn’s disease (CD) and ulcerative colitis (UC), is a chronic, relapsing inflammatory disorder of the gastrointestinal tract that significantly impacts patients’ quality of life (QoL), defined as overall physical, psychological, and social wellbeing (1, 2). IBD is characterized by alternating periods of active inflammation and remission. Some of the symptoms of IBD include abdominal pain, diarrhea, bleeding from the rectum, weight loss, and systemic effects such as fatigue, anemia, and nutritional deficiencies. These symptoms burden the disease and aid clinical diagnosis. Hemoglobin, albumin, CRP, and ESR are used to assess disease activity, systemic inflammation, and nutritional status in IBD patients in addition to clinical symptoms. Anemia and malnutrition may cause low hemoglobin and albumin levels, whereas increased CRP and ESR indicate inflammation (3). Since the disorder affects physical, emotional, and social health, comprehensive treatment is necessary. IBD is becoming more common worldwide. In 2023, 29.2 per 100,000 individuals worldwide had IBD. Based on age-standardized prevalence. Therefore, the burden of illness will continue to rise (4, 5). Especially in urbanizing areas where individuals live differently. IBD is spreading across Asia, notably in China. Urban China had 10.04 IBD cases per 100,000 person-years in 2016 (6). This highlights the need for effective and sustainable management strategies in the region. Genetic variations, family history, and environmental exposures, especially consumption of ultra-processed foods, contribute to elevated IBD risk. Chronic inflammation may be influenced by abnormal immune responses to gut bacteria, and microbial imbalances have been observed in patients with higher disease activity (7, 8). Chronic inflammation in IBD is driven in part by an abnormal immune response to gut microbiota, and microbial imbalances may exacerbate disease activity (7, 8).

Remission, prevention of complications, improved nutrition, and enhanced QoL are key treatment goals in IBD management. Standard treatments for severe or refractory cases include aminosalicylates, corticosteroids, immunomodulators, biologics, and surgery (9). Enteral nutrition (EN) is now a feasible adjuvant or primary treatment for CD. A nutritionally complete liquid formula is delivered orally or via feeding tubes as exclusive enteral nutrition (EEN) or as a meal supplement (10–12). EN delivers essential nutrients to malnourished individuals, thereby improving nutritional status, reducing intestinal inflammation, modulating gut microbiota, and supporting mucosal healing. EN has fewer systemic adverse effects than corticosteroids, making it a safer long-term treatment (13, 14). By addressing disease activity and nutritional deficiencies, EN may contribute to improvements in fatigue, appetite, and overall QoL. Given the rising prevalence of IBD in Asia and China and the therapeutic benefits of EN, its role in clinical care warrants careful evaluation. This study, therefore, examines the association between enteral nutrition and disease activity, nutritional status, laboratory indicators, and QoL in patients with IBD, including clinical outcomes, patient-reported improvements, and adherence.

## 2 Materials and methods

### 2.1 Ethical considerations

This study was conducted in accordance with the Declaration of Helsinki and relevant national regulations. This research was approved by the Ethics Committee of The Second Affiliated Hospital of Soochow University No. JD-HG-2025-087. The retrospective analysis used de-identified patient data; therefore, informed consent was waived. The institution’s ethical committee approved the trial, which kept patients anonymous.

### 2.2 Study design and setting

In a retrospective observational study, the use of EN and its association with clinical management, nutritional status and quality of life were evaluated in patients with IBD, including CD and UC. Data were collected at a single time point from hospital patient records and electronic medical records (EMR) from January 2022 to March 2025.

### 2.3 Study population and sample size

For eligibility, we screened 900 IBD patients. After applying inclusion/exclusion criteria and testing medical record completeness, 750 patients were evaluated (400 CD, 350 UC). Participants were aged ≥ 20 years with a confirmed diagnosis of CD or UC. Only oral or tube-fed patients were considered. Medical records must include demographics, illness features, laboratory testing, treatment history, and follow-up. QoL assessments using the Inflammatory Bowel Disease Questionnaire (15) were required for participation. Patients with celiac disease, gastrointestinal cancer, incomplete medical records, absence of EN, or advanced hepatic or renal disease were excluded, as these conditions could impair nutritional therapy or affect study outcomes. Additionally, patients with permanent colostomies or ileostomies were excluded, as surgical diversion may significantly alter nutrient absorption, bowel function, and disease activity assessment, potentially confounding the evaluation of enteral nutrition outcomes.

### 2.4 Data collection and outcome measures

Hospital EMRs and patient files were analyzed retrospectively. Demographics (age, gender, BMI), lifestyle factors (smoking, alcohol intake), comorbidities, disease characteristics (type, duration, severity), laboratory findings (hemoglobin, albumin, CRP, ESR), medication history, hospitalizations, surgical interventions, and complications were collected. Also documented were enteral nutrition (EN) therapy details such as formula type, caloric density, mode of administration, frequency, and duration. The validated 32-item Inflammatory Bowel Disease Questionnaire (IBDQ) assessed patient-reported outcomes in bowel symptoms, systemic symptoms, emotional function, social function, and general health/appetite (15). A pilot assessment with specialists and 30 IBD patients was conducted to ensure the IBDQ was contextually relevant, clear, and culturally appropriate, without altering its original scoring system. A panel of specialists and thirty IBD patients piloted the questionnaire to verify its construct and content validity. The reliability test shows strong internal consistency across all subdomains. As evidenced by Cronbach’s α value exceeding 0.85. For CD, remission was defined as a Crohn’s Disease Activity Index (CDAI) score < 150. For UC, remission was defined as a Partial Mayo Score ≤ 2, with no individual subscore > 1. Disease severity and response to enteral nutrition were evaluated using CDAI (for CD), Partial Mayo Score (for UC), and IBDQ total score improvement. As all pre- and post-intervention data were already documented in patient records, this study analyzed existing measurements to evaluate the effectiveness of EN without any additional interventions.

### 2.5 Statistical analysis

Data were analyzed using IBM SPSS Statistics, Version 26.0 (IBM Corp., Armonk, NY, United States). Clinical and laboratory measurements (like BMI, albumin, CRP, and ESR) documented before and after enteral nutrition (EN) in patient records were summarized and compared. Comparisons between independent groups (e.g., CD vs. UC) were performed using independent *t*-tests for continuous variables and Chi-square (χ^2^) tests for categorical variables. Continuous variables are reported as mean ± SD, and categorical variables as *n* (%). Results were considered statistically significant if *p* < 0.05.

## 3 Results and discussion

### 3.1 Demographic and clinical characteristics of the study population

Because this was a retrospective study, all clinical, laboratory, and nutritional data were obtained from existing patient medical records. For variables reported as changes after enteral nutrition (EN), pre-EN and post-EN values were extracted from routine clinical documentation recorded during standard patient care. A total of 750 patients with IBD were included, comprising 400 with CD and 350 with UC. Participants were aged from 20 to ≥60 years, with a mean age of 39.5 ± 10.8 years. Age was analyzed as a continuous variable in addition to categories. Statistical analysis showed no significant age difference between CD and UC (*p* = 0.42) (Table 1). Most patients (50.7%) were aged 30–49 years. The study population included 380 males (50.7%) and 370 females (49.3%) with a similar gender distribution CD (52.5%; men) and UC (48.6%; men) (*p* = 0.38). Participants had a mean BMI of 23.8 ± 3.5 kg/m^2^, with no significant difference between CD (23.5 ± 3.6) and UC (24.1 ± 3.4) (*p* = 0.12). The patient population was 8% underweight, 26.7% overweight, and 5.3% obese. Regarding lifestyle factors, 67% of patients were non-smokers, 24% were smokers, and 9.3% were ex-smokers. No significant difference was observed between the CD and UC groups (*p* = 0.46). Alcohol consumption was reported by 16% of participants, with no significant difference between groups (*p* = 0.28). Comorbidities included hypertension (13.3%), diabetes (10.7%), and other chronic conditions (6.7%), with no significant differences between CD and UC groups (*p* = 0.55).

**TABLE 1:** Demographic and baseline characteristics of study participants.

Characteristic	Total (*n* = 750)	Total%	CD (*n* = 400)	CD%	UC (*n* = 350)	UC%	*P*-value
Age (years)							0.42
20–29	120	16.0	70	17.5	50	14.3	–
30–39	180	24.0	100	25.0	80	22.9	–
40–49	200	26.7	110	27.5	90	25.7	–
50–59	150	20.0	70	17.5	80	22.9	–
≥60	100	13.3	50	12.5	50	14.3	–
Gender (*n*)							0.38
Men	380	50.7	210	52.5	170	48.6	–
Women	370	49.3	190	47.5	180	51.4	–
BMI (kg/m^2^, mean ± SD)	23.8 ± 3.5	–	23.5 ± 3.6	–	24.1 ± 3.4	–	0.12
Underweight (<18.5 kg/m^2^)	60	8.0	35	8.8	25	7.1	–
Normal weight (18.5–24.9 kg/m^2^)	450	60.0	230	57.5	220	62.9	–
Overweight (25–29.9 kg/m^2^)	200	26.7	110	27.5	90	25.7	–
Obese (≥30 kg/m^2^)	40	5.3	25	6.2	15	4.3	–
Smoking status (current/former/ non-smoker)							0.46
Non-smoker	500	66.7	260	65.0	240	68.6	–
Current smoker	180	24.0	100	25.0	80	22.9	–
Former smoker	70	9.3	40	10.0	30	8.6	–
Alcohol consumption n (%)							
	120	16.0	70	17.5	50	14.3	0.28
Comorbidities *n* (%)							0.55
Hypertension	100	13.3	50	12.5	50	14.3	–
Diabetes mellitus	80	10.7	40	10.0	40	11.4	–
Other chronic conditions	50	6.7	25	6.2	25	7.1	–

BMI, body mass index (kg/m^2^); CD, Crohn’s disease; UC, ulcerative colitis; SD, standard deviation. Percentages (%) are shown in separate columns, continuous variables as mean ± SD, categorical as *n* (%). Comparisons between CD and UC used independent *t*-test or Chi-square (χ^2^) test. *P* < 0.05 considered statistically significant.

### 3.2 Disease distribution and clinical characteristics

Table 2 shows the clinical characteristics of the study participants. It also shows the total illness rate of the research participants. Patients diagnosed with CD had a longer mean disease duration (6.5 ± 3.6 years) compared to those with UC (5.8 ± 3.4 years; *p* = 0.04). Disease severity was similar between the two groups, with 46.7% of patients in both groups classified as having moderate disease activity (*p* = 0.32). Hospitalization occurred in 42.5% of CD patients and 37.1% of UC patients, with no statistically significant difference (*p* = 0.18). Patients with CD had higher rates of surgical intervention (22.5%) compared with UC patients, and extraintestinal manifestations were also more frequent in CD (15% vs. 11.4%, *p* = 0.21). Laboratory parameters differed widely among groups. The study found that patients with CD had lower hemoglobin levels (11.2 ± 1.9 g/dL) compared to those with ulcerative colitis (11.8 ± 1.7 g/dL; *p* = 0.02). Contrarily, CRP and ESR levels were significantly higher (*p* < 0.001 and *p* < 0.01, respectively). CD patients showed lower blood albumin levels (3.6 ± 0.5 g/dL) compared to UC patients (4.0 ± 0.4 g/dL; *p* < 0.001). Regarding treatment patterns, CD patients were more likely to receive corticosteroids (37.5% vs. 28.6%, *p* = 0.01), immunomodulators (30% vs. 22.9%, *p* = 0.04), and biologic therapies (25% vs. 14.3%, *p* = 0.002) compared with UC patients. Complications were more common among CD patients, particularly fistula formation (12.5% vs. 2.9%, *p* < 0.001) and intestinal strictures (11.2% vs. 1.4%, *p* < 0.001). In contrast, Toxic megacolon was observed only among UC patients (2.9%, *p* = 0.005). Although not statistically significant (*p* = 0.25), nutritional deficiencies were slightly more prevalent in CD (17.5%) than in UC (14.3%). Among the total 750 IBD patients, 16% had nutritional deficiencies. The most common deficiencies were vitamin D (8%) and iron (6.7%), while vitamin B12 (2%) and folate (1.3%) deficiencies were less frequent.

**TABLE 2:** Disease distribution and clinical characteristics of study participants.

Characteristic	Total (*n* = 750)	CD (*n* = 400)	UC (*n* = 350)	*P*-value
Disease duration (years, mean ± SD)	6.2 ± 3.5	6.5 ± 3.6	5.8 ± 3.4	0.04*
Disease severity *n* (%)				0.32
Mild	280 (37.3)	150 (37.5)	130 (37.1)	
Moderate	350 (46.7)	180 (45)	170 (48.6)	
Severe	120 (16)	70 (17.5)	50 (14.3)	
Previous hospitalizations *n* (%)	300 (40)	170 (42.5)	130 (37.1)	0.18
Surgery history *n* (%)	150 (20)	90 (22.5)	60 (17.1)	0.09
Extraintestinal manifestations *n* (%)	100 (13.3)	60 (15)	40 (11.4)	0.21
Hemoglobin (g/dL)	11.5 ± 1.8	11.2 ± 1.9	11.8 ± 1.7	0.02*
CRP (mg/L)	12 ± 6	14 ± 7	10 ± 5	<0.001**
ESR (mm/hr)	25 ± 12	28 ± 13	22 ± 11	<0.01**
Albumin (g/dL)	3.8 ± 0.5	3.6 ± 0.5	4.0 ± 0.4	<0.001**
Corticosteroids use	250 (33.3)	150 (37.5)	100 (28.6)	0.01*
Immunomodulator	200 (26.7)	120 (30)	80 (22.9)	0.04*
Biologics	150 (20)	100 (25)	50 (14.3)	0.002**
Fistula	60 (8)	50 (12.5)	10 (2.9)	<0.001**
Stricture	50 (6.7)	45 (11.2)	5 (1.4)	<0.001**
Toxic megacolon	10 (1.3)	0 (0)	10 (2.9)	0.005**
Nutritional deficiencies *n* (%)				0.25
Iron deficiency	50 (6.7)	30 (7.5)	20 (5.7)	0.41
Vitamin D deficiency	60 (8)	35 (8.8)	25 (7.1)	0.46
Vitamin B12 deficiency	15 (2)	8 (2)	7 (2)	0.80
Folate deficiency	10 (1.3)	5 (1.2)	5 (1.4)	0.87

Data are presented as mean ± standard deviation (SD) or number (percentage). Comparisons between Crohn’s disease and ulcerative colitis groups were performed using the independent sample *t*-test for continuous variables and the chi-square test for categorical variables. Nutritional deficiencies assessed include iron deficiency (serum ferritin < 30 ng/mL), vitamin D deficiency (serum 25-hydroxyvitamin D < 20 ng/mL), vitamin B12 deficiency (<200 pg/mL), and folate deficiency (<3 ng/mL). A *p*-value < 0.05 was considered statistically significant. Significant results are denoted as follows: *p < 0.05, **p < 0.01.

### 3.3 Type and formulation of enteral nutrition administered

In this investigation, EN type and formulation varied between CD and UC subgroups in IBD patients (Table 3). Polymeric or standard formulas were most commonly utilized (40%), followed by semi-elemental or peptide-based formulas (33.3%) and elemental or amino acid-based formulas (26.7%). CD and UC groups did not vary statistically (*p* = 0.18). Elemental diets were prescribed more in CD (30%) than UC (22.9%). The mean caloric density of EN formulations was similar between groups (1.2 ± 0.1 kcal/mL, *p* = 0.65). Daily caloric intake was higher in CD patients (1850 ± 240 kcal/day) compared with UC patients (1750 ± 260 kcal/day, *p* = 0.01), and protein intake was also higher in CD (85 ± 16 g/day) than UC (75 ± 14 g/day, *p* < 0.001). Regarding EN delivery routes, oral or sip feeding was most common (53.3%), followed by nasogastric tube feeding (33.3%) and percutaneous endoscopic gastrostomy (13.3%), with no significant difference between groups (*p* = 0.27). The mean duration of EN therapy was similar (6 ± 2 weeks, *p* = 0.89), and the majority of patients (73.3%) received intermittent or bolus feeding, while 26.7% received continuous infusion (*p* = 0.56).

**TABLE 3:** Type and formulation of enteral nutrition administered.

Parameter/feature	Total (*n* = 750)	CD (*n* = 400)	UC (*n* = 350)	*P*-value
Type of EN formula				0.18
Polymer/standard formula *n* (%)	300 (40)	150 (37.5)	150 (42.9)	
Elemental/amino acid-based *n* (%)	200 (26.7)	120 (30)	80 (22.9)	
Semi-elemental/peptide-based *n* (%)	250 (33.3)	130 (32.5)	120 (34.3)	
Caloric density (kcal/mL, mean ± SD)	1.2 ± 0.1	1.2 ± 0.1	1.2 ± 0.1	0.65
Daily caloric intake (kcal/day, mean ± SD)	1800 ± 250	1850 ± 240	1750 ± 260	0.01*
Protein content (g/day, mean ± SD)	80 ± 15	85 ± 16	75 ± 14	<0.001**
Route of administration				0.27
Oral/sip feed *n* (%)	400 (53.3)	210 (52.5)	190 (54.3)	
Nasogastric tube *n* (%)	250 (33.3)	140 (35)	110 (31.4)	
Percutaneous endoscopic gastrostomy (PEG) *n* (%)	100 (13.3)	50 (12.5)	50 (14.3)	
Duration of EN therapy (weeks, mean ± SD)	6 ± 2	6 ± 2	6 ± 2	0.89
Frequency				0.56
Continuous *n* (%)	200 (26.7)	110 (27.5)	90 (25.7)	
Intermittent/bolus *n* (%)	550 (73.3)	290 (72.5)	260 (74.3)	

Data are presented as mean ± standard deviation (SD) or number (percentage). Comparisons between Crohn’s disease and ulcerative colitis groups were performed using the independent sample *t*-test for continuous variables and the chi-square test for categorical variables. A *p*-value < 0.05 was considered statistically significant. Significant results are denoted as follows: *p < 0.05, **p < 0.01.

### 3.4 Impact of enteral nutrition on clinical, nutritional, laboratory, and quality of life outcomes

Based on retrospective review of patient records, enteral nutrition (EN) was associated with improvements in clinical remission, nutritional parameters, inflammatory markers, and quality of life among 750 IBD patients (CD = 400, UC = 350). Approximately 60% of patients achieved remission, with CD patients showing a marginally higher proportion than UC patients. Fewer disease flares were observed in 66.7% of patients, similar between CD (67.5%) and UC (65.7%; *p* = 0.64) (Table 4). Medication usage declined modestly: 13.3% required fewer corticosteroids, and 16% used fewer immunomodulators or biologics (*p* > 0.05). Mean BMI increased by + 1.2 ± 0.8 kg/m^2^ (CD: + 1.3 ± 0.9; UC: + 1.1 ± 0.7; *p* = 0.07). Serum albumin increased significantly (CD: + 0.35 ± 0.2 g/dL; UC: + 0.25 ± 0.2 g/dL; *p* = 0.01). Hemoglobin improved (*p* = 0.03). Iron deficiency in 6.7% and vitamin D deficiency in 8% were corrected. Inflammatory markers: CRP decreased by 5 ± 3 mg/L (CD: −6 ± 3; UC: −4 ± 2.5; *p* = 0.04). ESR decreased by 8 ± 5 mm/h (*p* = 0.03). Quality of life measured using IBDQ improved, with mean total scores rising to 160 ± 20, without significant differences between CD and UC (*p* = 0.12). Adverse events were minimal: only 8% experienced moderate gastrointestinal symptoms, including nausea, diarrhea, and bloating (CD: 8.8%, UC: 7.1%; *p* = 0.48).

**TABLE 4:** Changes in clinical, nutritional, laboratory, and quality-of-life outcomes before and after enteral nutrition based on retrospective medical record review.

Outcome/parameter	Total (*n* = 750)	CD (*n* = 400)	UC (*n* = 350)	*P*-value
Clinical outcomes				
Clinical remission (%)	450 (60%)	250 (62.5%)	200 (57.1%)	0.18
Reduction in flare-ups (%)	500 (66.7%)	270 (67.5%)	230 (65.7%)	0.64
Need for corticosteroids post-EN (%)	100 (13.3%)	60 (15%)	40 (11.4%)	0.19
Medication reduction (%)	120 (16%)	70 (17.5%)	50 (14.3%)	0.31
Nutritional status				
BMI change (kg/m^2^, mean ± SD)	+1.2 ± 0.8	+1.3 ± 0.9	+1.1 ± 0.7	0.07
Albumin change (g/dL, mean ± SD)	+0.3 ± 0.2	+0.35 ± 0.2	+0.25 ± 0.2	0.01*
Hemoglobin change (g/dL, mean ± SD)	+0.8 ± 0.6	+0.9 ± 0.7	+0.7 ± 0.5	0.03*
Iron deficiency resolved (%)	50 (6.7%)	30 (7.5%)	20 (5.7%)	0.41
Vitamin D normalized (%)	60 (8%)	35 (8.8%)	25 (7.1%)	0.46
Inflammatory markers				
CRP reduction (mg/L, mean ± SD)	−5 ± 3	−6 ± 3	−4 ± 2.5	0.04*
ESR reduction (mm/h, mean ± SD)	−8 ± 5	−9 ± 5	−7 ± 4	0.03*
QoL (IBDQ total)	160 ± 20	162 ± 18	158 ± 22	0.12
Adverse effects (overall)	60 (8%)	35 (8.8%)	25 (7.1%)	0.48

Values are expressed as mean ± standard deviation (SD) or percentage (%). Comparisons between Crohn’s disease (CD) and ulcerative colitis (UC) groups were conducted using independent *t*-tests for continuous variables and chi-square tests for categorical variables. Statistical significance was set at *p* < 0.05. * Indicate statistically significant differences between CD and UC groups.

### 3.5 Multivariate regression analysis

Using retrospectively collected clinical and laboratory data from 750 IBD patients treated with EN, multivariable regression identified independent predictors of clinical remission and change in quality of life (ΔIBDQ). Patients adhering to EN exhibited higher odds of remission and greater gains in QoL as measured by the IBDQ score (β = 7.5 points, 95% CI 4.3–10.7, *p* < 0.001). Extended EN duration (>6 weeks) correlated with improved QoL, though it did not significantly predict clinical remission. Disease severity was a negative predictor: Moderate disease: lower odds of remission (OR 0.72, 95% CI 0.52–0.99, *p* = 0.045) and reduced ΔIBDQ (β = −3.0, 95% CI −5.8 to −0.2, *p* = 0.035). Severe disease: even lower odds of remission (OR 0.50, 95% CI 0.32–0.78, *p* = 0.002) and greater reduction in ΔIBDQ (β = −5.5, 95% CI −9.2 to −1.8, *p* = 0.004). Major complications (fistula, stricture, toxic megacolon) reduced the likelihood of remission and tended to worsen QoL outcomes. Laboratory predictors: Patients with baseline hypoalbuminemia (<3.5 g/dL) had increased chances of remission and greater improvements in QoL. Hemoglobin < 11 g/dL and CRP > 10 mg/L were not independent predictors. Other factors such as age, gender, BMI, disease type, disease duration, type or route of enteral nutrition, caloric or protein intake, smoking, alcohol use, and comorbidities—did not significantly affect clinical remission or quality-of-life improvement (Table 5).

**TABLE 5:** Multivariate regression analysis of predictors of clinical remission and quality of life improvement in Inflammatory Bowel Disease (IBD) patients receiving enteral nutrition.

Predictor	Clinical remission (OR, 95% CI)	*P*-value	ΔIBDQ score (β, 95% CI)	*P*-value
Demographics				
Age 20–39 (ref)	1	–	0	–
Age 40–59	0.95 (0.68–1.32)	0.75	−0.8 (−3.2 to 1.6)	0.50
Age 60+	0.78 (0.52–1.17)	0.23	−2.5 (−6.0 to 1.0)	0.16
Gender (female vs. male)	1.15 (0.88–1.51)	0.32	1.9 (−1.2 to 4.9)	0.23
BMI (ref)				
Underweight	0.81 (0.55–1.19)	0.28	−1.8 (−5.0 to 1.4)	0.27
Overweight	1.02 (0.73–1.44)	0.89	0.5 (−2.3 to 3.3)	0.72
Obese	0.88 (0.51–1.51)	0.63	−1.0 (−5.0 to 3.0)	0.63
Disease characteristics				
Disease type (Crohn vs. UC)	1.22 (0.92–1.63)	0.17	2.5 (−0.5 to 5.5)	0.10
Disease duration > 5 years	0.91 (0.69–1.20)	0.50	−0.6 (−3.0 to 1.8)	0.63
Disease severity				
Moderate vs. mild	0.72 (0.52–0.99)	0.045	−3.0 (−5.8 to −0.2)	0.035
Severe vs. mild	0.50 (0.32–0.78)	0.002	−5.5 (−9.2 to −1.8)	0.004
Extraintestinal manifestations (yes vs. no)	0.85 (0.59–1.21)	0.36	−1.5 (−4.2 to 1.2)	0.27
Surgery history (yes vs. no)	0.88 (0.62–1.25)	0.47	−1.2 (−4.0 to 1.6)	0.40
Complications (fistula/stricture/toxic megacolon)	0.61 (0.39–0.95)	0.03	−3.5 (−7.0 to 0.0)	0.05
Laboratory parameters				
Baseline CRP > 10 mg/L	0.92 (0.67–1.26)	0.61	−1.2 (−3.8 to 1.4)	0.36
Baseline albumin < 3.5 g/dL	1.45 (1.05–2.00)	0.02	2.0 (0.5–3.5)	0.008
Hemoglobin < 11 g/dL	0.85 (0.60–1.21)	0.37	−1.8 (−4.5 to 0.9)	0.19
Enteral nutrition characteristics				
EN adherence (fully vs. partial/non)	2.15 (1.49–3.10)	< 0.001	7.5 (4.3–10.7)	<0.001
ENType (elemental vs. polymeric)	1.08 (0.78–1.48)	0.63	1.5 (−1.0 to 4.0)	0.24
EN Route (tube vs. oral)	0.88 (0.63–1.22)	0.44	−1.5 (−4.0 to 1.0)	0.23
Duration of EN therapy > 6 weeks	1.05 (0.97–1.14)	0.21	0.6 (0.2–1.0)	0.003
Daily caloric intake > 1,800 kcal	1.12 (0.82–1.52)	0.48	1.0 (−1.5 to 3.5)	0.43
Protein intake > 80 g/day	1.08 (0.79–1.47)	0.63	1.2 (−1.2 to 3.6)	0.32
Lifestyle factors				
Smoking (current vs. non)	0.79 (0.56–1.11)	0.17	−2.0 (−4.5 to 0.5)	0.11
Alcohol use (yes vs. no)	0.85 (0.58–1.25)	0.41	−1.8 (−4.8 to 1.2)	0.24
Comorbidities				
Hypertension	0.92 (0.64–1.32)	0.66	−0.5 (−3.0 to 2.0)	0.69
Diabetes	0.87 (0.60–1.27)	0.48	−1.0 (−4.0 to 2.0)	0.52
Other chronic conditions	0.90 (0.56–1.45)	0.67	−0.8 (−4.0 to 2.4)	0.61

Multivariable analyses were performed using logistic regression to identify predictors of *clinical remission* (dependent variable: remission = yes/no) and linear regression to evaluate determinants of *change in quality of life (*Δ*IBDQ)*, defined as post-EN IBDQ score minus baseline IBDQ score. Results are presented as odds ratios (OR) for remission and regression coefficients (β) for ΔIBDQ, along with their 95% confidence intervals (CI). A *p*-value < 0.05 was considered statistically significant. Hypoalbuminemia was defined as serum albumin < 3.5 g/dL; patients with albumin ≥ 3.5 g/dL served as the reference category.

## 4 Discussion

The present study found that most patients with IBD were aged 30–49 years, which is consistent with previous epidemiological studies reporting that IBD frequently manifests during early and middle adulthood (16, 17). The nearly equal gender distribution observed in this study is also in agreement with global reports indicating no strong sex predominance in IBD (18), although some Asian studies have reported a slightly higher prevalence of CD among males (19). It was observed that the majority of individuals in this study had normal BMI. Weight loss has been connected to IBD, particularly CD, but current research implies a move toward normal weight and overweight. This “hidden obesity” in IBD patients may be linked to diet, lifestyle, and illness management changes (20–22). Alcohol may potentially alter gut microbiota and worsen IBD symptoms in some patients (23, 24). IBD may also be linked with metabolic diseases like hypertension and diabetes. Chronic systemic inflammation causes metabolic imbalances and cardiometabolic illnesses in this population (25, 26). It was also observed that CD patients had a longer disease duration and more difficulties than UC patients, suggesting a more complex and aggressive trajectory. Previous research has shown that Crohn’s disease causes transmural inflammation, fistulas, and strictures (27, 28). High CRP, ESR, and low hemoglobin and albumin linked with nutritional impairment and systemic inflammation in CD patients. These findings support prior findings that CD patients have higher inflammation and nutritional deficits than UC patients. The study found that CD patients utilize more corticosteroids, immunomodulators, and biologic treatments, supporting the idea that CD requires comprehensive treatment. Studies reveal Crohn’s disease patients need more advanced treatments due to its difficult path (29). It was also observed that toxic megacolon was only found in UC patients, supporting prior observations that this illness is a serious consequence of UC (30, 31). These data show CD and UC’s different clinical features. UC is colon-specific inflammation, while CD has systemic and structural consequences. EN formulation and administration were similar for CD and UC patients, but CD patients got more elemental formulations and higher caloric and protein support, possibly due to small intestinal involvement’s larger nutritional and metabolic demands (32, 33). Previous study observed that elemental and semi-elemental formulae improve mucosal healing and inflammation control, notably in CD, which may explain their higher use in this subgroup (34, 35). Most patients preferred oral or sip feeding, consistent with current evidence associating oral EN to improved compliance in mild-to-moderate disease, while nasogastric or tube feeding is related with low oral intake or severe disease (36). Previous research found that intermittent or bolus feeding was more prevalent than continuous infusion for ambulatory patients due to higher tolerability, feasibility, and flexibility (37, 38). These findings support the role of EN as an effective nutritional and anti-inflammatory adjunct in IBD. CD patients often received higher nutritional support and elemental formulations, which may be linked to more severe disease activity. Improvements in BMI, albumin, hemoglobin, and inflammatory markers were observed, reflecting associations between EN and enhanced nutritional status and reduced systemic inflammation. Prior studies have reported that EN is associated with reduced corticosteroid dependence, improved remission rates, and decreased disease activity indices, particularly in CD (39). EN’s therapeutic effects are thought to be linked with modulation of the gut microbiota, improved intestinal barrier function, and anti-inflammatory effects, thereby supporting mucosal healing and systemic recovery. Observed improvements in BMI, albumin, and hemoglobin align with previous reports showing restoration of protein-energy status after 6–8 weeks of EN therapy (40, 41). Similarly, reductions in CRP and ESR are associated with EN’s anti-inflammatory role via downregulation of pro-inflammatory cytokines and restoration of intestinal homeostasis (42). Although some studies report greater mucosal healing and quality-of-life improvements in CD, this study observed comparable benefits in UC patients, albeit slightly less pronounced, which may be linked to disease-specific pathophysiology, EN adherence, or tolerance. The low incidence of adverse effects validates EN as a safe and well-tolerated supportive therapy in both CD and UC (43, 44). Regression analysis indicated that adherence to EN and treatment duration were associated with better clinical and quality-of-life outcomes in IBD patients. Severe disease and major complications (fistula, strictures, toxic megacolon) were linked with limited EN response, consistent with reports that patients with complicated IBD often require surgery or biologic escalation. Patients with baseline hypoalbuminemia (<3.5 g/dL) showed greater improvement after EN therapy. Hypoalbuminemia in IBD often reflects protein-energy malnutrition, systemic inflammation, and impaired intestinal absorption. Nutritional therapy may therefore have a larger measurable impact in these patients by restoring protein balance, reducing inflammatory activity, and improving intestinal barrier function. Consequently, individuals with lower baseline albumin may demonstrate greater clinical and quality-of-life improvements following nutritional support (45). However, improvement in albumin levels or clinical outcomes does not necessarily translate into reduced mortality. Serum albumin is widely recognized as a marker of disease severity and systemic inflammation rather than a direct causal factor in survival outcomes. Therefore, although patients with lower baseline albumin may show greater nutritional and clinical improvements after EN, mortality risk may remain influenced by underlying disease activity, complications, and comorbid conditions. Other factors, including demographics, disease type, EN formula, route, and caloric/protein intake, were not independently associated with outcomes, suggesting that adherence and disease-specific characteristics are stronger predictors of therapeutic benefit (45, 46). Demographics, disease type, EN formula, route, and caloric/protein intake did not alter outcomes, suggesting that adherence and disease-specific factors better predict therapeutic success. Higher adherence and longer duration of EN therapy were associated with increased clinical remission and improved quality of life, while severe baseline disease and structural difficulties had less effect. These findings highlight the need for personalized nutritional therapy in IBD management and targeted interventions for nutritionally at-risk individuals.

## 5 Limitations

This study has several limitations. Its retrospective, single-center design limits causal inference and generalizability. The absence of a control group, variations in EN formulations, duration, and administration routes, as well as reliance on medical records for adherence, introduce potential bias and confounding. Incomplete or inconsistent data, non-standardized follow-up intervals, and concurrent therapies (e.g., corticosteroids, immunomodulators, biologics) further complicate attribution of outcomes solely to enteral nutrition. Despite multivariate regression, residual confounding from unmeasured factors such as treatment changes and baseline disease severity may persist. Prospective, multicenter randomized trials are needed to confirm these findings and clarify the independent effect of EN.

## 6 Conclusion

In this retrospective study based on routinely documented clinical records, the effects of EN on clinical, nutritional, inflammatory, and quality-of-life outcomes were evaluated in patients with IBD. The study observed that EN was associated with improved clinical remission, enhanced nutritional status, and reduced inflammation. It was observed that Patients receiving EN therapy exhibited better body composition, higher levels of protein and hemoglobin, and lower levels of inflammation. It was also observed that the quality of life increased with a few side effects. Longer EN duration increased adherence, which improved remission rates and health outcomes. EN appears to be a safe, effective, and nutritionally supportive complement to normal therapy, with CD patients benefiting most. EN may also improve pharmacological treatment efficacy, resulting in more consistent IBD management.

## Data Availability

The original contributions presented in this study are included in this article/supplementary materials, further inquiries can be directed to the corresponding author.
